# Printed colorimetric chemosensor array on a 96-microwell paper substrate for metal ions in river water

**DOI:** 10.3389/fchem.2023.1134752

**Published:** 2023-02-23

**Authors:** Yui Sasaki, Xiaojun Lyu, Tsuyoshi Minami

**Affiliations:** Institute of Industrial Science, The University of Tokyo, Tokyo, Japan

**Keywords:** pattern recognation, chemosensor array, metal ion, molecular self-assembly, imaging analysis, paper

## Abstract

Here, we propose a printed 96-well microtiter paper-based chemosensor array device (PCSAD) to simultaneously detect metal ions for river water assessment. Colorimetric chemosensors for metal ions have been designed based on molecular self-assembly using off-the-shelf catechol dyes and a phenylboronic acid (PBA) derivative. The colorimetric self-assembled chemosensors consisting of catechol dyes and a PBA derivative on a 96-well microtiter paper substrate demonstrated various color changes according to the disassembly of the ensembles by the addition of nine types of metal ions. An in-house-made algorithm was used to automate imaging analysis and extract color intensities at seven types of color channels from a captured digital image, allowing for rapid data processing. The obtained information-rich inset data showed fingerprint-like colorimetric responses and was applied to the qualitative and quantitative pattern recognition of metal ions using chemometric techniques. The feasibility of the 96-well microtiter PCSAD for environmental assessment has been revealed by the demonstration of a spike-and-recovery test against metal ions in a river water sample.

## Introduction

With increasing attention paid to high-throughput chemical analysis, computational methods are widely utilized owing to their powerful data processability (Jurs et al., 2000; Anzenbacher et al., 2010; Li et al., 2019). In this regard, chemical sensor platforms for the simultaneous detection of multiple analytes, which are referred to as sensor arrays (Dickinson et al., 1996; Lavigne et al., 1998; Vlasov and Legin, 1998; Rakow and Suslick, 2000), have been vigorously developed thus far. Chemosensor arrays are analytical tools for pattern recognition at the molecular levels, which enable the visualization of molecular recognition information through optical changes upon capturing analytes (Kitamura et al., 2009; Peveler et al., 2016; Sasaki et al., 2021a). Based on the inherent cross-reactivities of artificial receptors (Gardner, 1991), chemosensors in an array display a fingerprint-like response pattern according to the different types of analytes and their concentrations (Anzenbacher et al., 2010; Sasaki et al., 2021a). Therefore, qualitative and quantitative chemical information at the molecular level can be analyzed by data processing of the optical fingerprint-like response pattern (Huang et al., 2013; Sener et al., 2014; Xu et al., 2014; Hwang et al., 2015), allowing for accurate sensing, even in real samples (Palacios et al., 2008; Li J. et al., 2020; Li Y. et al., 2020; Bowyer et al., 2022).

Molecular recognition information on chemosensor arrays in solution is conventionally acquired by spectrophotometers, whereas the applicability to on-site analysis is limited by solution-based sensing systems (Sasaki et al., 2021b; Sasaki et al., 2022). Hence, we have focused on chemosensor arrays embedded in solid-state sensor devices to establish easy-to-use chemical sensor devices (Xu and Bonizzoni, 2020; Lyu et al., 2021; Zhang et al., 2021; Lyu et al., 2022). Paper is a representative solid-state substrate in the field of analytical chemistry, and its superior processability has facilitated the development of printed paper-based analytical devices (Martinez et al., 2010; Feng et al., 2013; Yamada et al., 2015; Karita and Kaneta, 2016; Tan et al., 2022). Disposable paper-based analytical devices have been applied to real-sample analysis (e.g., river water) in combination with portable recording apparatuses for environmental assessment (Chen et al., 2014; Karita and Kaneta, 2016; Kung et al., 2019; Liu et al., 2019), while simultaneous discrimination of multiple metal ions and quantitative detection by solid-state portable sensor devices in river water samples is still at the frontier. Thus far, we designed a paper-based chemosensor array device (PCSAD) for the simultaneous detection of multiple metal ions based on molecular self-assembly.

**FIGURE 1:**
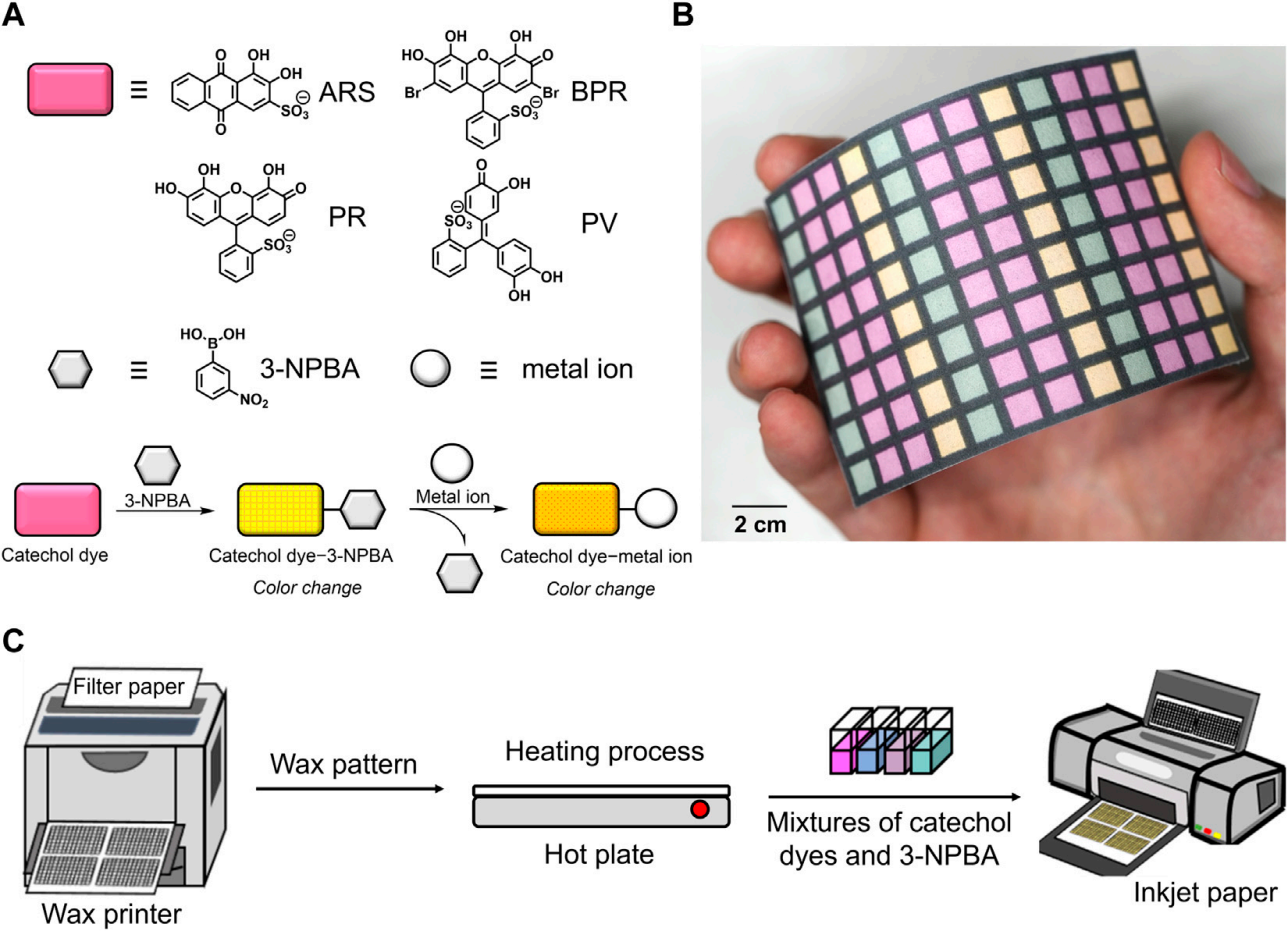
**(A)** Chemical structures of the building blocks for the self-assembled chemosensors and the schematic colorimetric detection mechanisms for metal ions. **(B)** Photograph of the 96-well microtiter PCSAD. **(C)** Schematic of the PCSAD fabrication.

The ability of chemosensor arrays to perform classification and prediction relies on the quality of the inset data, although data processing using powerful statistical methods can be applied. This indicates that an optimal design of chemosensors is required to obtain an information-rich data matrix for pattern recognition (Sasaki et al., 2020; Sasaki et al., 2021a). Such an information-rich dataset is conventionally acquired by collecting various types of chemosensors possessing different optical characteristics to increase the variables that correspond to wavelength, absorbance, and fluorescence intensity. Considering contribution of each chemosensor to pattern recognition (Liu et al., 2013), the sophisticated design of chemosensors that provide drastic optical changes is more important than the number of chemosensors for obtaining information-rich datasets (Sasaki et al., 2020). A molecular self-assembled system through non-covalent bonds exhibits inherent reversible behavior based on the assembly and disassembly of building blocks. By utilizing the unique features of self-assemblies to chemosensors, the optical responses of the chemosensors can be manipulated through competitive responses among the building blocks of the chemosensors and analytes (Nguyen and Anslyn, 2006; Sedgwick et al., 2021). In particular, the optical contrast derived produced by the reversible molecular self-assemblies is significant for imaging analysis on the PCSADs. This indicates that chemosensor designs based on non-covalent chemistry are promising for establishing the PCSADs for real-sample analysis. Phenylboronic acid (PBA) forms a boronate ester with *cis*-diols, and the attractive reversibility of PBA derivatives can be utilized as a manipulator of a catechol dye for tuning optical properties (Kubo et al., 2005; Sasaki et al., 2017). The optical profiles of catechol dyes are manipulated by boronate esterification (Springsteen and Wang, 2001; Tomsho and Benkovic, 2012) causing blueshifts because of the changes in the negative charge states of the catechol dyes at neutral pH conditions (Tomsho and Benkovic, 2012; Sasaki et al., 2017). Upon the addition of the target metal ions to the complex of the catechol dye and PBA derivative, colorimetric changes are induced by the release of the PBA derivative from the boronate esters accompanied by the complexation of the catechol dyes and metal ions (Kaushik et al., 2015; Lin et al., 2018). The coordination between the catechol dyes and target metal ions promotes deprotonation of the catechol dyes (Zhang et al., 2007), whereby redshifts can be observed (Sasaki et al., 2017). In this study, four types of catechol dyes [i.e., alizarin red S (ARS), bromopyrogallol red (BPR), pyrogallol red (PR), and pyrocatechol violet (PV)] and 3-nitrophenylboronic acid (3-NPBA) (Sasaki et al., 2019) were employed as the building blocks for the colorimetric chemosensors against nine metal ions (i.e., Cu^2+^, Zn^2+^, Ni^2+^, Cd^2+^, Al^3+^, Pb^2+^, Co^2+^, Ca^2+^, and Mg^2+^) (Figure 1). In this regard, the nitro group of 3-NPBA enhances the Lewis-acidity of boronic acid, facilitating boronate esterification with catechol dyes under neutral pH conditions (Sasaki et al., 2017; Sasaki et al., 2019). For example, boron−nitrogen (B−N) interactions have been widely used to form boronate esters with diol units, while a single B−N interaction alone was insufficient to tune the color profiles of dyes through complexation with PBA derivatives (Koumoto et al., 1998). Specifically, the role of the electron-withdrawing nitro group is more significant than that of the B-N interaction (Koumoto et al., 1998).

During the device fabrication, an office wax printer was used to dispense the hydrophobic barrier onto the filter paper to avoid leakage of the target solutions (Figure 1). Chemosensor solutions made of 3-NPBA and catechol dyes were injected into a 96-well microtiter PCSAD using an office inkjet printer to obtain solid-state detection portions (Figure 1). Upon drop-casting the metal ion solutions onto each well, various color changes were observed and rapidly scanned using a flatbed scanner. The obtained digital color images were automatically analyzed by an in-house-made algorithm, followed by the construction of a data matrix containing color intensities in different color channels. We evaluated the detectability of the manufactured 96-well microtiter PCSAD and further applied it to the regression analysis of metal ions in a river water sample using pattern recognition techniques.

## Materials and methods

### Reagents

All reagents used for device fabrication and sensing were utilized without further pre-treatment. ARS, a commercially available river sample with trace elements in river water (Elevated Level, NMIJ CRM 7202-c, Supplementary Table S1), and iron(III) nitrate enneahydrate (Fe^3+^) were obtained from FUJIFILM Wako Pure Chemical Co., Ltd. The target metal ions including cobalt(II) perchlorate hexahydrate (Co^2+^), calcium perchlorate tetrahydrate (Ca^2+^), lead(II) perchlorate trihydrate (Pb^2+^), cadmium perchlorate hydrate (Cd^2+^), nickel(II) perchlorate hexahydrate (Ni^2+^), copper(II) perchlorate hexahydrate (Cu^2+^), magnesium perchlorate hexahydrate (Mg^2+^), aluminum perchlorate non-ahydrate (Al^3+^), mercury(II) perchlorate hydrate (Hg^2+^), and zinc perchlorate hexahydrate (Zn^2+^) were purchased from Sigma-Aldrich. The building blocks of the chemosensors purchased from Tokyo Chemical Industry Co., Ltd. were 3-NPBA, BPR, PR, and PV. A buffer material purchased from DOJINDO was 4-(2-hydroxyethyl)-1-piperazineethanesulfonic acid (HEPES). All aqueous solutions were prepared using Milli-Q water (18.2 Ω cm).

### Device fabrication

Based on our previous reports on the colorimetric self-assembled chemosensor arrays using off-the-shelf materials (Sasaki et al., 2017) and the fabrication of paper-based sensor devices (Sasaki et al., 2021b; Lyu et al., 2021), a colorimetric PCSAD for metal ions was designed. A filter paper adjusted to A4 size (Whatman grade 1, GE Healthcare, Buckinghamshire, UK) was used as the solid-state substrate for PCSAD. Hydrophobic barriers were fabricated on the smooth front side of the filter paper using an office wax printer (ColorQube 8580, Xerox). A 96-well plate pattern was printed with a square shape (5 mm^2^ × 5 mm^2^), and the gap between each well was set to 2 mm. The printed pattern was annealed at 150°C for 60 s on a hot plate (NHS-450ND, NISSIN Co.). The printed wax melts during the annealing process, and hydrophobic barriers were formed by the penetration of the melted wax into the filter paper. The filter paper substrate was cooled to 25°C. The self-assembled complexes, which were evaluated using a UV-vis spectrophotometer in a previous study (Sasaki et al., 2017), were applied to printing inks for device fabrication in this assay. Therefore, a HEPES solution (50 mM, pH 7.4) containing catechol dyes (2 mM) and 3-NPBA (30 mM) was dispensed into the wells with five printing cycles using an inkjet printer (PIXUS TS203, Canon Inc.). Moreover, the target metal ions contained in the HEPES buffer solution (50 mM) or the mixture of river water and HEPES (1:1, v/v) (10 μL) adjusted to pH 7.4 were pipetted onto each well and then dried at room temperature under dark conditions. The optical responses of the 96-well microtiter PCSAD were quickly recorded using a flatbed scanner (CanoScan 9000F Mark II, Canon Inc.) with a scan resolution of 600 dpi.

### Data acquisition and analysis

An in-house-made algorithm constructed using MathWorks MATLAB 2022 was applied to the imaging analysis of the captured images of the color response. The entire image was first automatically divided into image slices that contained two control areas and ten repetition areas, while the wax backgrounds (i.e., black area in the PCSAD) were eliminated by the morphological detection using the red channel. Thus, the 96-wells of the PCSAD were individually separated for further imaging analyses. In this analysis, the color responses on the wells with added metal ions (i.e., the sensing area) were evaluated based on the colorimetric differences between the sensing and control areas (i.e., wells without added metal ions). A data matrix was constructed using the color intensities of the four chemosensors at seven optical channels with RGB (i.e., red, green, and blue color channels), grayscale, and YC_b_C_r_ (i.e., Y for the luma component and, C_b_ and C_r_ for blue- and red-difference chroma components), indicating 28 variables. Further, Student’s *t*-test was used to remove two outliers among the ten repetitions for each metal ion. Linear discriminant analysis (LDA) for the qualitative and semi-quantitative assays was performed using SYSTAT 13 without any further data treatment. Support vector machine (SVM) with Solo 9.0. performed a quantitative analysis of spike-and-recovery tests. The mean relative error values of the multisensory system (Oleneva et al., 2019) was evaluated to determine the limit of detection (LOD).

## Results and discussion

**FIGURE 2:**
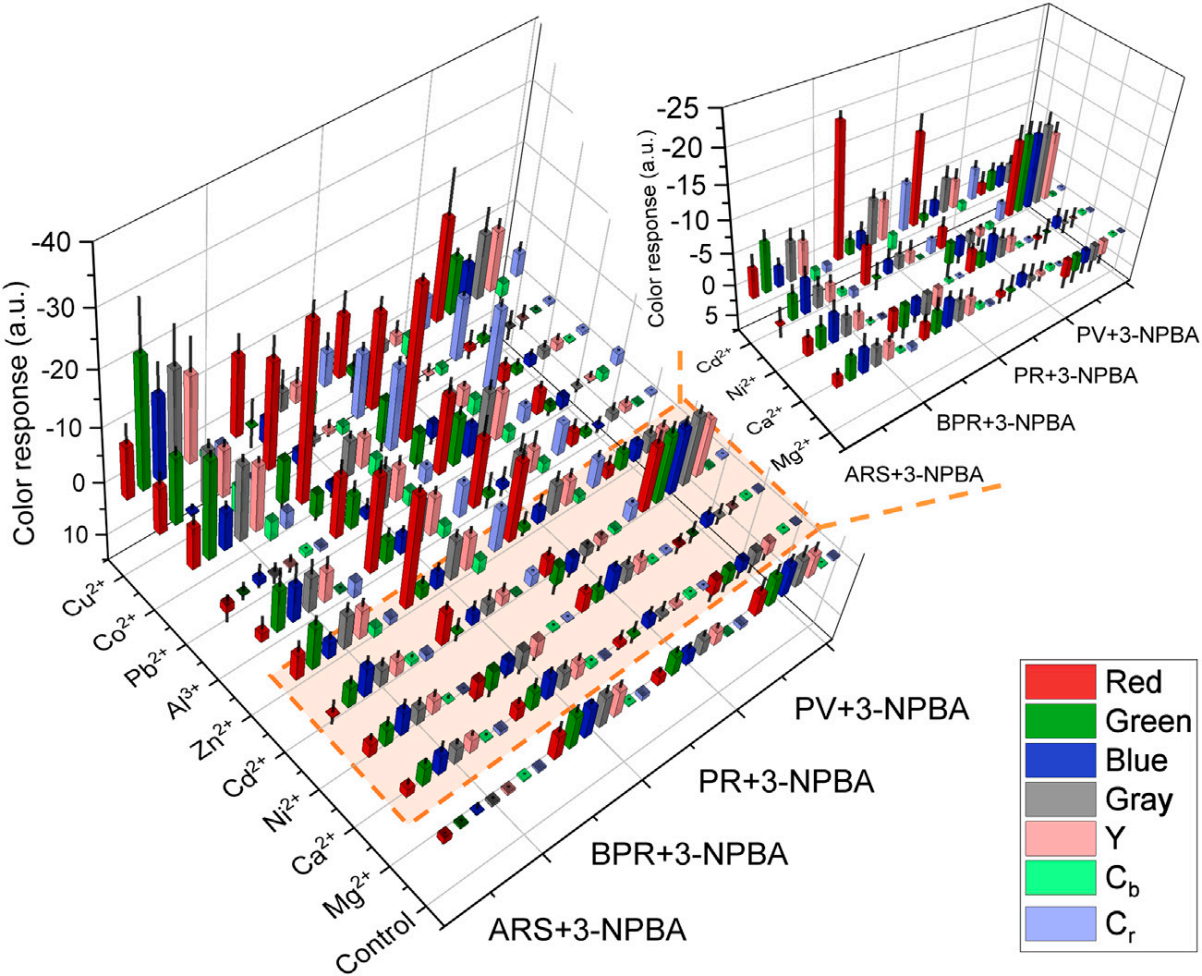
Color response profile of the 96-well microtiter PCSAD upon adding target metal ions (50 μM) in a HEPES buffer solution (50 mM) at pH 7.4.

The 96-well microtiter PCSAD exhibited various color changes with different types of metal ions, which stemmed from the different binding affinities of the catechol dyes to the analytes (Kubo et al., 2005; Sasaki et al., 2017). As shown in Figure 2, the color intensity profile of the 96-well microtiter PCSAD obtained by imaging analysis demonstrated a fingerprint-like colorimetric response pattern against the nine metal ions, implying its high potential for pattern recognition-driven chemosensing. Therefore, a paper-based chemosensor array system was applied to the simultaneous discrimination of nine types of metal ions using LDA, enabling the classification of clusters and decreasing the multi-dimensional inset data for pattern recognition (Anzenbacher et al., 2010).

**FIGURE 3:**
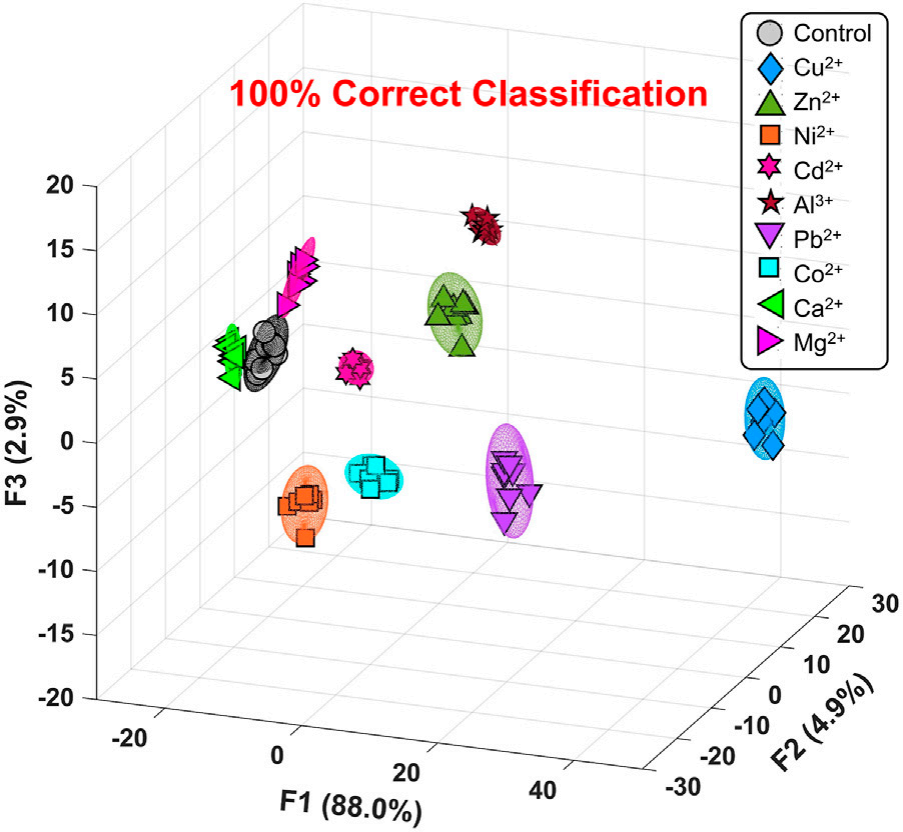
Canonical score plot by LDA for the qualitative assay against nine types of metal ions (50 μM) in a HEPES buffer solution (50 mM, at pH 7.4) with 95% confidence ellipsoids. All clusters with eight repetitive datasets for each analyte were distributed with 100% correct classification.

**FIGURE 4:**
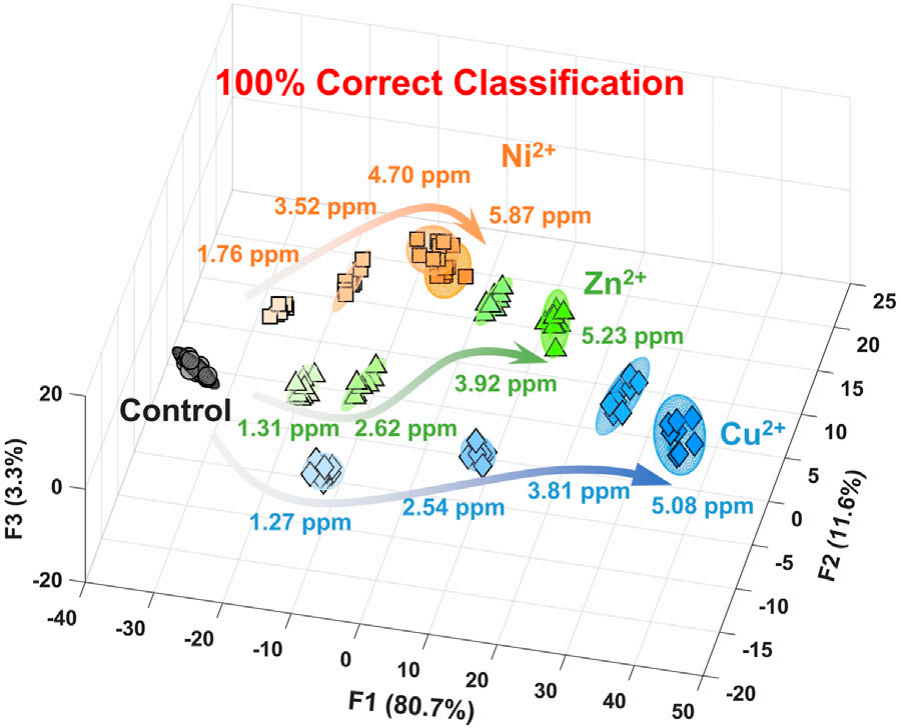
Canonical score plot by LDA for the semi-quantitative assay against Ni^2+^, Cu^2+^, and Zn^2+^ in a HEPES buffer solution (50 mM, at pH 7.4) with 95% confidence ellipsoids. All clusters with eight repetitive datasets for each analyte were distributed with 100% correct classification.

Figure 3 and Supplementary Figure S1 show the canonical score plot containing ten clusters (nine metal ions and a control) with eight repetition datasets for each analyte. The LDA result suggested the success of the discrimination of nine metal ions without misclassification (Supplementary Table S1). In contrast, the PCSAD unsuccessfully discriminated Fe^3+^ and Hg^2+^, which was probably due to the hydrolysis of the metal ions at neutral pH conditions. Moreover, the printed 96-well microtiter PCSAD was used to perform a semi-quantitative assay to simultaneously evaluate the discrimination power of analytes and their concentrations. The selected three species (i.e., Ni^2+^, Cu^2+^, and Zn^2+^) could have an antagonistic effect on the growth of microorganisms in ecosystems, whereby the quantitative detection of the levels of the three metal ions is essential from the perspective of environmental assessment (Payette and Yamamoto, 2011). The LDA results in Figure 4 show a concentration-dependent cluster distribution for all the analytes, indicating the accuracy of the manufactured 96-well microtiter PCSAD (Supplementary Table S2). Moreover, the LoD of the 96-well PCSAD for Cu^2+^, Zn^2+^, and Ni^2+^ was determined to be 0.15, 0.17, and 0.28 ppm, respectively (Supplementary Figure S3). The estimated values represented the sufficient detectability judging from the values of environmental quality standards (Verschoor et al., 2011).

**FIGURE 5:**
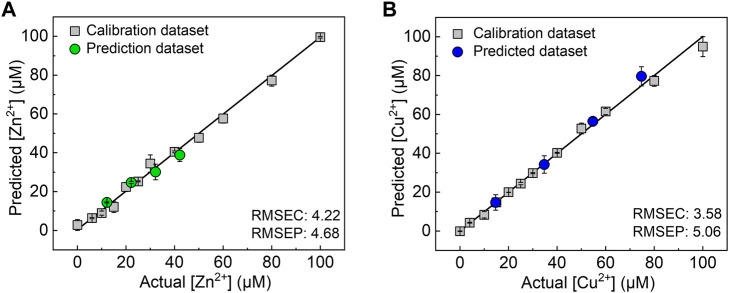
Results of the SVM regression for **(A)** Zn^2+^ and **(B)** Cu^2+^ in the river water sample. The values of the root-mean-square errors of calibration (RMSEC) and prediction (RMSEP) suggest the accuracy of the established model and prediction results.

Finally, we demonstrated a spike and recovery test for Ni^2+^, Cu^2+^, and Zn^2+^ in a river water sample using SVM, which is a powerful chemometric algorithm for building a linear calibration line in a complicated response pattern (Hamel, 2009; Minami et al., 2012). The calibration datasets (gray squares) for each target metal ion were respectively obtained in 0–100 µM range of the target metal ions in a HEPES buffer (50 mM, pH 7.4). As shown in Figure 5, the prediction datasets for Zn^2+^ (green circles) and Cu^2+^ (blue circles) are correctly distributed on the calibration lines with a low root-mean-square error of prediction. However, the unknown concentrations of Ni^2+^ in the river water samples were not successfully predicted (Supplementary Figure S4) because of the weaker response of the 96-well microtiter PCSAD to Ni^2+^ than to Cu^2+^ and Zn^2+^ as shown in the color intensity profile (Figure 2). Furthermore, the recovery rates for Zn^2+^ at 10, 20, and 30 µM and Cu^2+^ at 20, 40, and 60 µM were estimated to be 89%–125% (Supplementary Table S4). The proposed PCSAD combined with pattern recognition accurately predicted unknown concentrations of metal ions in the river water samples, suggesting the usability of the proposed method for the accurate and sensitive detection of metal ions in environmental samples.

## Conclusion

In this study, we designed a colorimetric chemosensor array on a 96-well microtiter paper-based device for metal ion detection. The self-assembled chemosensor array was designed to obtain a unique optical contrast upon the addition of metal ions, which was derived from a competitive assay. In this regard, the reversibility of the colorimetric boronate esters contributed to an increase in the optical response patterns during the assembly and disassembly with catechol dyes. Indeed, chemosensors comprising catechol dyes and 3-NPBA as printing inks demonstrated various color changes upon the addition of nine metal ions, allowing pattern recognition-driven chemical sensing through the fingerprinting-like colorimetric response on paper. Hence, the 96-well microtiter PCSAD succeeded in the simultaneous discrimination of nine metal ions with 100% correct classification. In the semi-quantitative assay of Ni^2+^, Cu^2+^, and Zn^2+^, a concentration-dependent cluster distribution was observed using LDA. Finally, the applicability of the 96-well microtiter PCSAD for metal ion assessment in river water was evaluated using a spike-and-recovery test. The estimated recovery rates for metal ions in the river water sample (89%–125%) suggest its potential as a facile paper-based analytical device enabling both qualitative and quantitative analysis of real samples. Although further improvements in the accuracy of the sensor devices are required, we believe that an appropriate sensor design based on molecular recognition chemistry could create new avenues for the realization of supramolecular analytical devices.

## Data Availability

The original contributions presented in the study are included in the article/Supplementary Material, further inquiries can be directed to the corresponding author.
