# Supplementary material for: Printed colorimetric chemosensor array on a 96-microwell paper substrate for metal ions in river water
Source: Front Chem. 2023 Feb 23;11:1134752. doi: 10.3389/fchem.2023.1134752 (PMC9996040; doi:10.3389/fchem.2023.1134752)
Supplement: Supplementary file 1 [file DataSheet1.pdf]

*Supplementary Material*

# Printed colorimetric chemosensor array on a 96-microwell paper substrate for metal ions in river water

**Yui Sasaki,† Xiaojun Lyu,† and Tsuyoshi Minami***

Institute of Industrial Science, The University of Tokyo, 4-6-1 Komaba, Meguro-ku, Tokyo, 153-8505, Japan

*** Correspondence:**
Tsuyoshi Minami
tminami@g.ecc.u-tokyo.ac.jp

†These authors contributed equally to this work.

## Contents

**1. Qualitative assay 2**

**2. Semi-quantitative assay 3**

**3. Spike-and-recovery test for metal ions in river water 4**

# 1. Qualitative assay

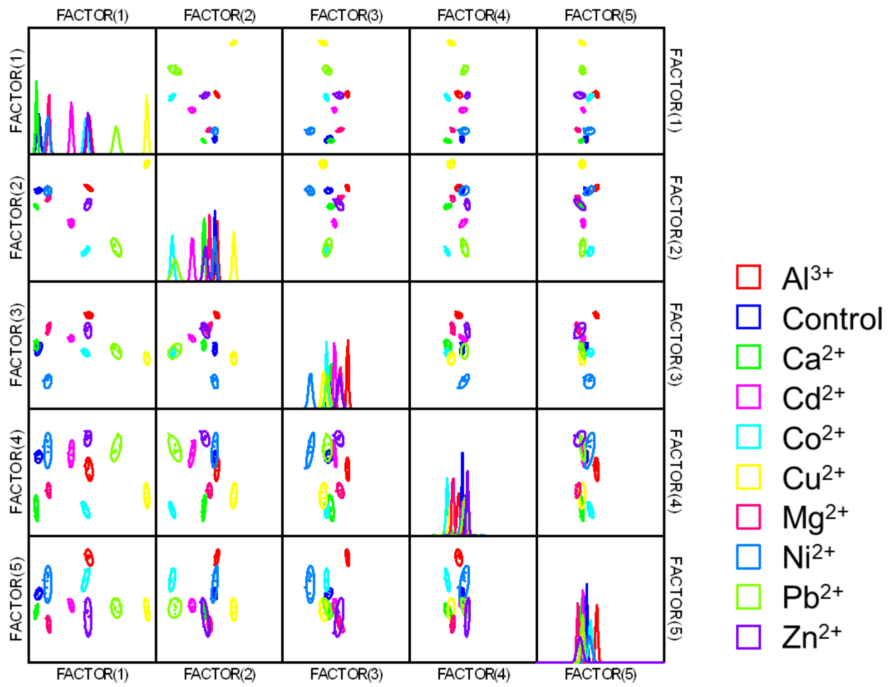


**Supplementary Figure 1.** Canonical score plot by LDA for the qualitative assay against the nine types of metal ions.

**Supplementary Table 1**. Jackknifed classification matrix of the qualitative assay against the nine types of metal ions

| | $Al^{3+}$ | Control | $Ca^{2+}$ | $Cd^{2+}$ | $Co^{2+}$ | $Cu^{2+}$ | $Mg^{2+}$ | $Ni^{2+}$ | $Pb^{2+}$ | $Zn^{2+}$ | %correct |
|---|---|---|---|---|---|---|---|---|---|---|---|
| $Al^{3+}$ | 8 | 0 | 0 | 0 | 0 | 0 | 0 | 0 | 0 | 0 | 100 |
| Control | 0 | 8 | 0 | 0 | 0 | 0 | 0 | 0 | 0 | 0 | 100 |
| $Ca^{2+}$ | 0 | 0 | 8 | 0 | 0 | 0 | 0 | 0 | 0 | 0 | 100 |
| $Cd^{2+}$ | 0 | 0 | 0 | 8 | 0 | 0 | 0 | 0 | 0 | 0 | 100 |
| $Co^{2+}$ | 0 | 0 | 0 | 0 | 8 | 0 | 0 | 0 | 0 | 0 | 100 |
| $Cu^{2+}$ | 0 | 0 | 0 | 0 | 0 | 8 | 0 | 0 | 0 | 0 | 100 |
| $Mg^{2+}$ | 0 | 0 | 0 | 0 | 0 | 0 | 8 | 0 | 0 | 0 | 100 |
| $Ni^{2+}$ | 0 | 0 | 0 | 0 | 0 | 0 | 0 | 8 | 0 | 0 | 100 |
| $Pb^{2+}$ | 0 | 0 | 0 | 0 | 0 | 0 | 0 | 0 | 8 | 0 | 100 |
| $Zn^{2+}$ | 0 | 0 | 0 | 0 | 0 | 0 | 0 | 0 | 0 | 8 | 100 |
| Total | 8 | 8 | 8 | 8 | 8 | 8 | 8 | 8 | 8 | 8 | 100 |

## 2. Semi-quantitative assay

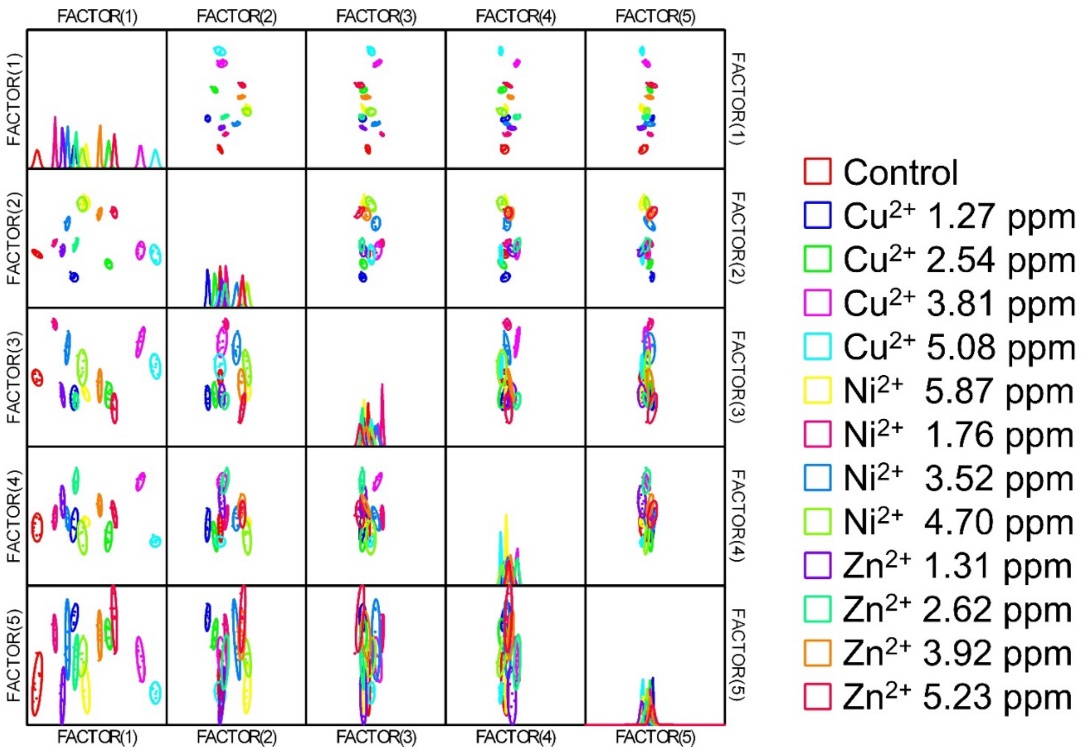


**Supplementary Figure 2.** Canonical score plot by LDA for the semi-quantitative assay.

**Supplementary Table 2**. Jackknifed classification matrix of the semi-quantitative assay

| | Control | $Cu^{2+}$ 1.27 ppm | $Cu^{2+}$ 2.54 ppm | $Cu^{2+}$ 3.81 ppm | $Cu^{2+}$ 5.08 ppm | $Ni^{2+}$ 1.76 ppm | $Ni^{2+}$ 3.52 ppm | $Ni^{2+}$ 4.70 ppm | $Ni^{2+}$ 5.87 ppm | $Zn^{2+}$ 1.31 ppm | $Zn^{2+}$ 2.62 ppm | $Zn^{2+}$ 3.92 ppm | $Zn^{2+}$ 5.23 ppm | %correct |
|---|---|---|---|---|---|---|---|---|---|---|---|---|---|---|
| Control | 8 | 0 | 0 | 0 | 0 | 0 | 0 | 0 | 0 | 0 | 0 | 0 | 0 | 100 |
| $Cu^{2+}$ 1.27 ppm | 0 | 8 | 0 | 0 | 0 | 0 | 0 | 0 | 0 | 0 | 0 | 0 | 0 | 100 |
| $Cu^{2+}$ 2.54 ppm | 0 | 0 | 8 | 0 | 0 | 0 | 0 | 0 | 0 | 0 | 0 | 0 | 0 | 100 |
| $Cu^{2+}$ 3.81 ppm | 0 | 0 | 0 | 8 | 0 | 0 | 0 | 0 | 0 | 0 | 0 | 0 | 0 | 100 |
| $Cu^{2+}$ 5.08 ppm | 0 | 0 | 0 | 0 | 8 | 0 | 0 | 0 | 0 | 0 | 0 | 0 | 0 | 100 |
| $Ni^{2+}$ 1.76 ppm | 0 | 0 | 0 | 0 | 0 | 8 | 0 | 0 | 0 | 0 | 0 | 0 | 0 | 100 |
| $Ni^{2+}$ 3.52 ppm | 0 | 0 | 0 | 0 | 0 | 0 | 8 | 0 | 0 | 0 | 0 | 0 | 0 | 100 |
| $Ni^{2+}$ 4.70 ppm | 0 | 0 | 0 | 0 | 0 | 0 | 0 | 8 | 0 | 0 | 0 | 0 | 0 | 100 |
| $Ni^{2+}$ 5.87 ppm | 0 | 0 | 0 | 0 | 0 | 0 | 0 | 0 | 8 | 0 | 0 | 0 | 0 | 100 |
| $Zn^{2+}$ 1.31 ppm | 0 | 0 | 0 | 0 | 0 | 0 | 0 | 0 | 0 | 8 | 0 | 0 | 0 | 100 |
| $Zn^{2+}$ 2.62 ppm | 0 | 0 | 0 | 0 | 0 | 0 | 0 | 0 | 0 | 0 | 8 | 0 | 0 | 100 |
| $Zn^{2+}$ 3.92 ppm | 0 | 0 | 0 | 0 | 0 | 0 | 0 | 0 | 0 | 0 | 0 | 8 | 0 | 100 |
| $Zn^{2+}$ 5.23 ppm | 0 | 0 | 0 | 0 | 0 | 0 | 0 | 0 | 0 | 0 | 0 | 0 | 8 | 100 |
| Total | 8 | 8 | 8 | 8 | 8 | 8 | 8 | 8 | 8 | 8 | 8 | 8 | 8 | 100 |

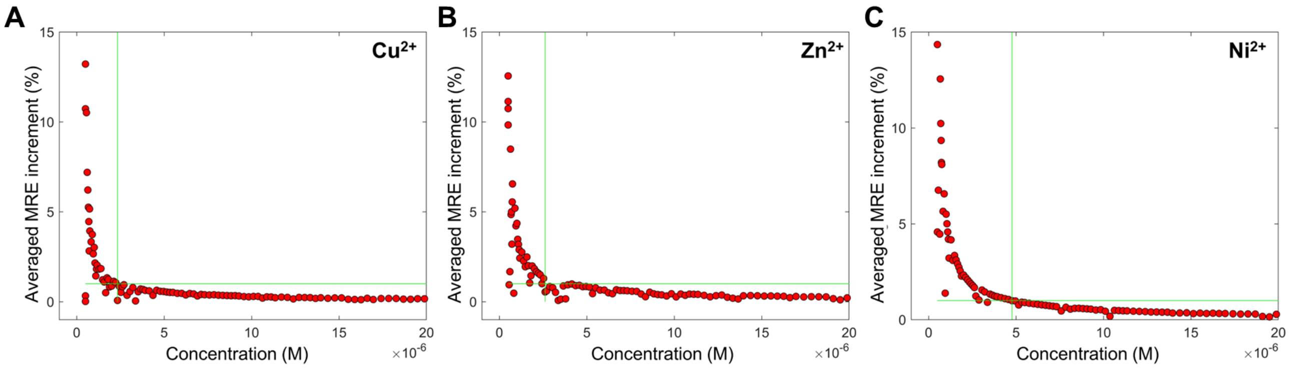


**Supplementary Figure 3.** LoD estimation for (A) $Cu^{2+}$, (B) $Zn^{2+}$and (C) $Ni^{2+}$ in the HEPES buffer at pH 7.4. The concentration region corresponding to the average mean relative error (MRE) increment from 0 to 15% is shown. The horizontal green line indicates the maximal acceptable magnitude of MRE fluctuations at 99% confidence. The vertical green line indicated the LoD value. The results showed the 96-well microtiter PCSAD reached the LoD for $Cu^{2+}$, $Zn^{2+}$and $Ni^{2+}$ at 0.15, 0.17 and 0.28 ppm, respectively.

## 3. Spike-and-recovery test for metal ions in river water

**Supplementary Table 3**. The elements of the river water sample

| Element | Concentration | Element | Concentration | Element | Concentration |
|---|---|---|---|---|---|
| B | 44.0 μg/kg | Zn | 10.6 μg/kg | Sb | 0.0095 μg/kg |
| Al | 21.8 μg/kg | As | 1.17 μg/kg | Ba | 5.74 μg/kg |
| Cr | 5.16 μg/kg | Se | 1.03 μg/kg | Pb | 1.018 μg/kg |
| Mn | 5.04 μg/kg | Rb | 0.653 μg/kg | Na | 3.68 mg/kg |
| Fe | 27.1μg/kg | Sr | 33.5 μg/kg | Mg | 1.26 mg/kg |
| Ni | 1.06 μg/kg | Mo | 0.183 μg/kg | K | 0.836 mg/kg |
| Cu | 10.1 μg/kg | Cd | 1.01 μg/kg | Ca | 4.59 mg/kg |

**Supplementary Table 4**. The result of the spike-and-recovery test for $Cu^{2+}$ and $Zn^{2+}$ in river water

| Metal Ion | Added (μM) | Founded (μM) | Recovery (%) |
|---|---|---|---|
| $Cu^{2+}$ | 20 | 19.80 ± 4.48 | 97 |
| | 40 | 41.78 ± 2.22 | 104 |
| | 60 | 64.94 ± 4.94 | 108 |
| $Zn^{2+}$ | 10 | 12.52 ± 0.52 | 125 |
| | 20 | 17.98 ± 4.02 | 90 |
| | 30 | 26.71 ± 3.29 | 89 |

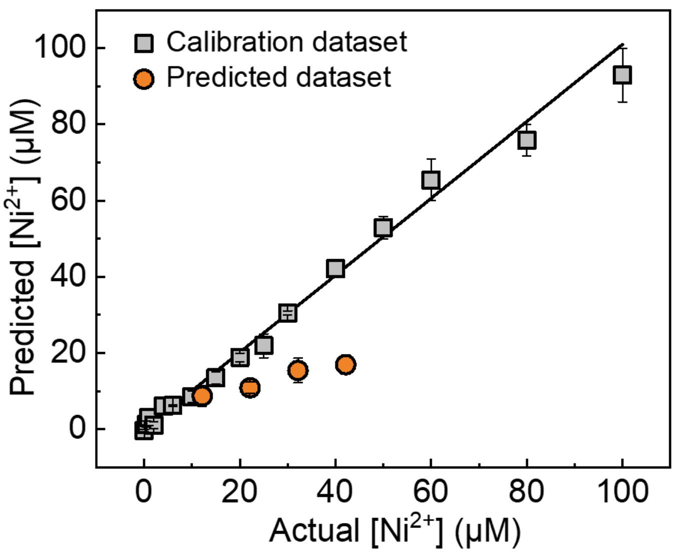


**Supplementary Figure 4.** Result of the SVM regression for $Ni^{2+}$ in the river water sample.
